# Supplementary material for: Cell-type-specific representation of spatial context in the rat prefrontal cortex
Source: iScience. 2024 Apr 15;27(5):109743. doi: 10.1016/j.isci.2024.109743 (PMC11070673; doi:10.1016/j.isci.2024.109743)
Supplement: Document S1. Figures S1–S5 [file mmc1.pdf]

iScience, Volume *27*

Supplemental information

Cell-type-specific representation of spatial context in the rat prefrontal cortex

Hans Brünner, Hoseok Kim, Sofie Ährlund-Richter, Josina Anna van Lunteren, Ana Paula Crestani, Konstantinos Meletis, and Marie Carlén

FIGURE S1

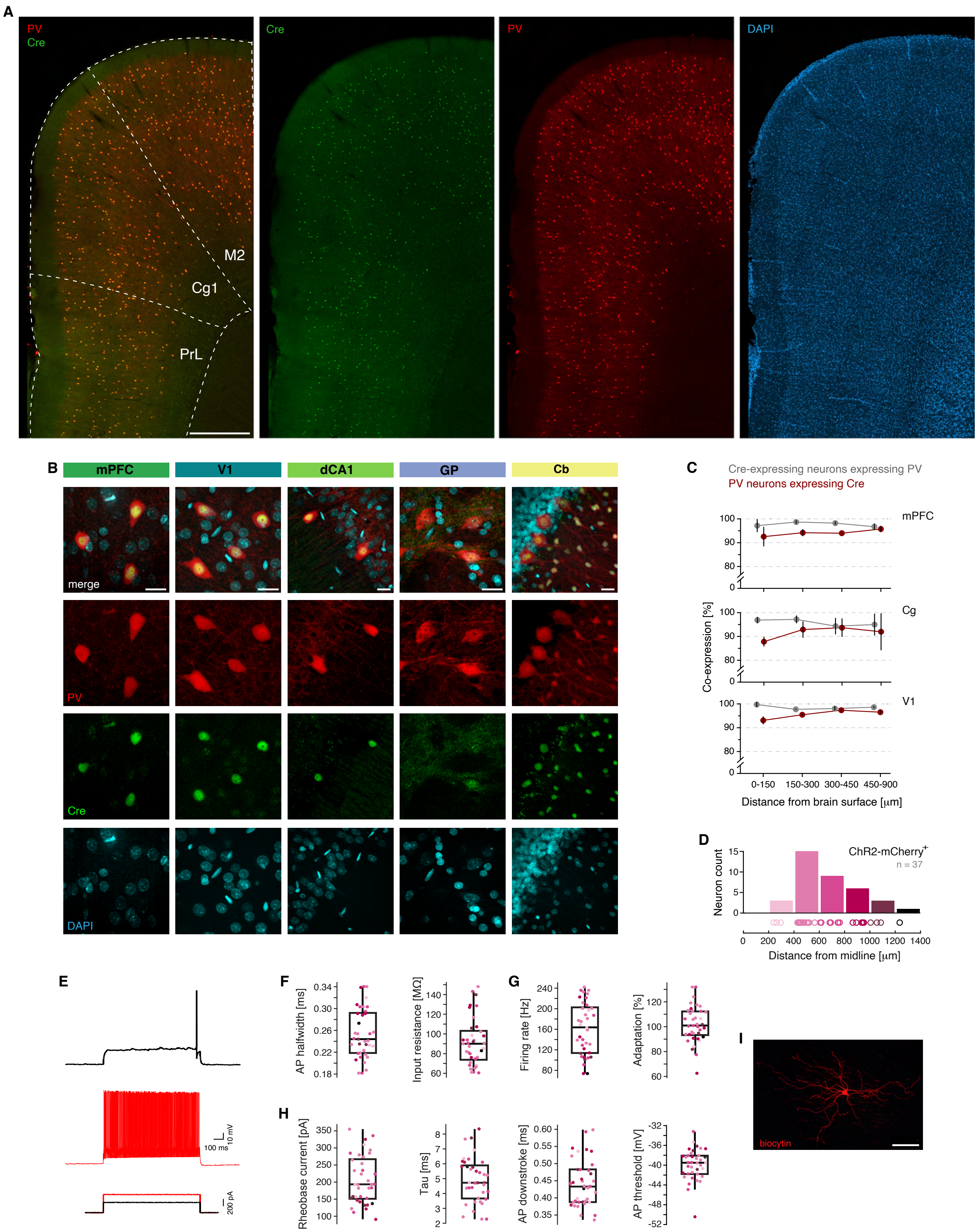
A
PV
Cre
M2
Cg1
PrL
Cre
PV
DAPI
B
mPFC
V1
dCA1
GP
Cb
merge
PV
Cre
DAPI
C
Cre-expressing neurons expressing PV
PV neurons expressing Cre
mPFC
Cg
V1
Co-expression [%]
0-150
150-300
300-450
450-900
Distance from brain surface [μm]
D
ChR2-mCherry+
n = 37
Neuron count
Distance from midline [μm]
E
100 ms
10 mV
200 pA
F
AP halfwidth [ms]
Input resistance [MΩ]
G
Firing rate [Hz]
Adaptation [%]
H
Rheobase current [pA]
Tau [ms]
AP downstroke [ms]
AP threshold [mV]
I
biocytin

Figure S1. Additional characterization of the PV-Cre rat line, related to Figure 1.

(**A**) Example of immunohistochemical detection of Cre (green) in prefrontal PV neurons (red) in an adult PV-Cre rat. Nuclei counterstained with DAPI (cyan).

(**B**) Split channels of the immunohistochemical detection of Cre (green) in PV neurons (red) in the cerebrum and cerebellum of adult PV-Cre rats presented in Figure 1B. Nuclei counterstained with DAPI (cyan).

(**C**) Investigation of the specificity and efficiency, respectively, of targeting of Cre to PV neurons throughout the cortical depth in three different cortical regions (Cg, mPFC, and V1). Gray: specificity, i.e., the fraction Cre-expressing neurons also expressing PV. Red: efficiency, i.e., the fraction PV neurons also expressing Cre.

(**D**) The distribution across the cortical depth of the recombined (i.e., ChR2-mCherry expressing) mPFC PV neurons recorded with whole-cell patch-clamp electrophysiology ex vivo. Bin size: 200 µm.

(**E**-**H**) Recombined mPFC neurons (n = 37) in adult PV-Cre rats display a typical FS phenotype.

(**E**) Response of a recombined mPFC PV neuron to a 1 s current pulse at rheobase current (black) and at twice the rheobase current (red).

(**F**) Box plots of the AP halfwidth, and input resistance, respectively, of recombined mPFC PV neurons.

(**G**) Box plots of the AP firing rate, and adaptation, respectively, of recombined mPFC PV neurons.

(**H**) Box plots of additional intrinsic properties of the recombined mPFC PV neurons. Rheobase current: 206 ± 70 pA, time constant (tau): 4.9 ± 1.5 ms, AP downstroke: 0.44 ± 0.07 ms, AP threshold: -40 ± 3 mV (mean, s.d.).

(**I**) Representative example of the morphology of a recombined mPFC PV neuron filled with Biocytin.

Scale bars: 500 µm (A), 20 µm (B), 100 µm (I); mean ± SEM (C); boxplots (F-H): black bar: median, box: 25th-75th percentile, whiskers: 99.3% data coverage.

FIGURE S2

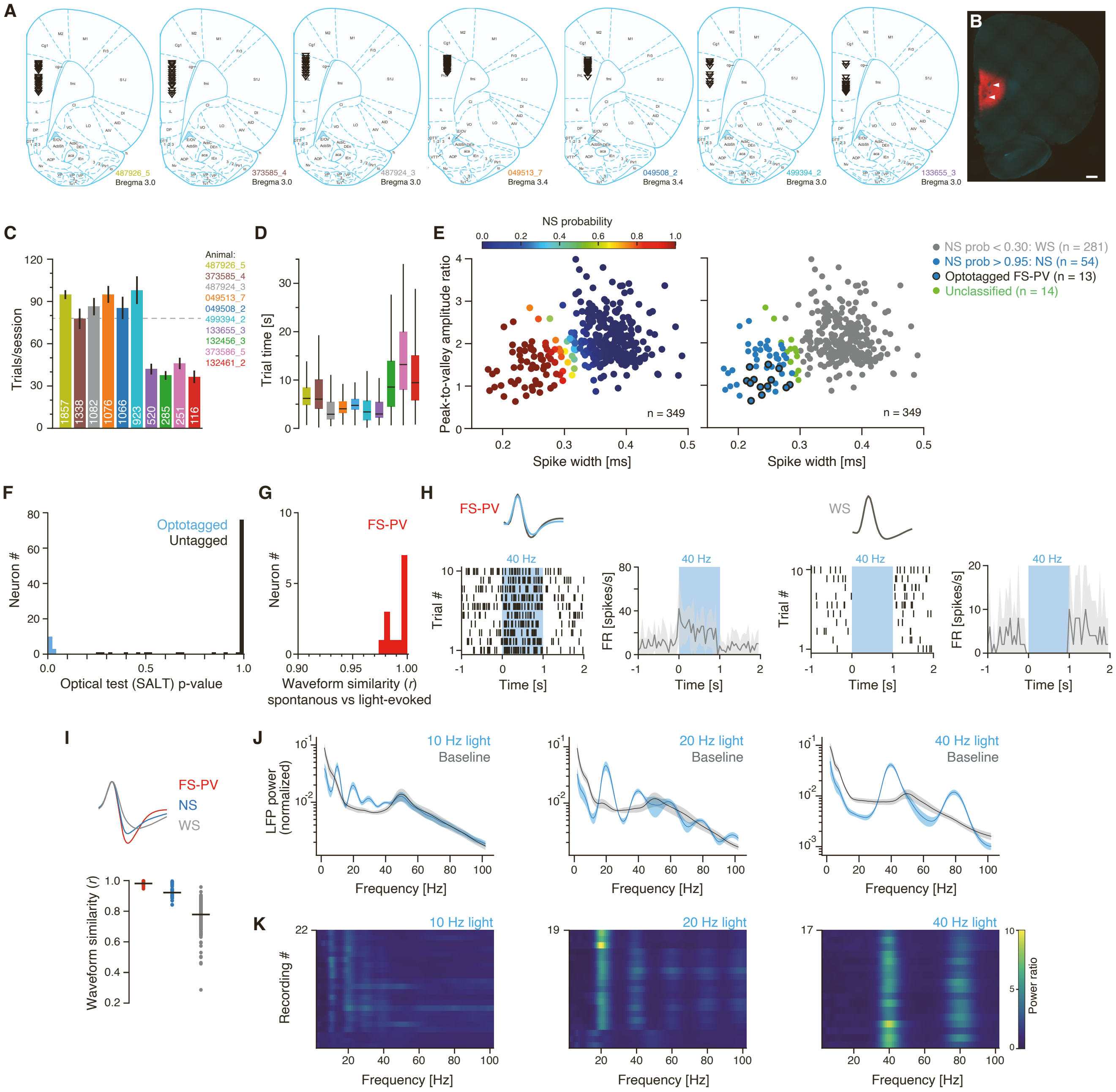

A
Bregma 3.0
Bregma 3.4
B
C
Trials/session
Animal:
487926_5
373585_4
487924_3
049513_7
049508_2
499394_2
133655_3
132456_3
373586_5
132461_2
D
Trial time [s]
E
NS probability
Peak-to-valley amplitude ratio
Spike width [ms]
n = 349
NS prob < 0.30: WS (n = 281)
NS prob > 0.95: NS (n = 54)
Optotagged FS-PV (n = 13)
Unclassified (n = 14)
F
Optotagged
Untagged
Neuron #
Optical test (SALT) p-value
G
FS-PV
Waveform similarity (r)
spontanous vs light-evoked
H
FS-PV
WS
40 Hz
Trial #
FR [spikes/s]
Time [s]
I
FS-PV
NS
WS
Waveform similarity (r)
J
10 Hz light
20 Hz light
40 Hz light
Baseline
LFP power (normalized)
Frequency [Hz]
K
Recording #
Power ratio
Frequency [Hz]

Figure S2. Electrophysiological recordings in awake PV-Cre rats: optotagging, induction of gamma oscillations, and behavior, related to Figure 1.

(**A**) Anatomical outline of the reconstructed recording sites in the PV-Cre rats (N = 7) included in the electrophysiological analyses.

(**B**) Example coronal section from one (133655_3) of the seven PV-Cre rats. Red: ChR2-mCherry expression in mPFC FS-PV neurons. White arrowheads: electrolytic lesions used for reconstruction of the last recording site.

(**C**-**D**) Detailed analysis of the behavior in the self-paced, goal-directed reward seeking task during electrophysiological recordings. 1 recording session: 100 trials, or max 1 h. Session time for implanted rats (N = 10): 24 ± 9 minutes/session.

(**C**) Trials per session (n = 78 ± 27), and the total number of trials (numbers in bars) for all implanted PV-Cre rats (N = 10). Four rats conducted a comparably low number of trials per session, and we therefore analyzed the temporal variability of the individual trials (D) to catch suboptimal trial behavior. Dashed line: mean number of trials per session across the animal cohort.

(**D**) Trial time, identifying highly varied trial behavior for three rats (132456_3 (green), 373586_5 (pink), and 132461_2 (red)). These rats in addition did few trials per session and < 500 trials in total (C) and were therefore excluded from electrophysiological analyses.

(**E**) Left: for objective classification of mPFC neurons into WS and NS, a gaussian mixture model (GMM) was fitted to the spike width and the peak-to-valley ratio (PVR) of the individual neurons. This revealed the NS probability (gradient bar) of individual neurons. Right: the classification of WS, and NS, neurons, respectively, based on the GMM clustering. Units with high NS probability (> 0.95) were classified as NS (n = 54; blue) and units with low NS probability (< 0.30) were classified as WS (n = 281; gray). Neurons with intermediate NS probability were not classified (n = 14; green) and discarded from future analysis. All optotagged FS-PV neurons (n = 13; black circles) as expected belonged to the NS population.

(**F**) Histogram of SALT p-values, identifying direct light-activation (optotagging) of 13 neurons (blue, $p < 0.05$; n = 102 analyzed neurons, recorded during optotagging sessions).

(**G**) Comparison of the spontaneous waveform (mean) to the light-evoked waveform (mean) of individual optotagged FS-PV neurons (n = 13), revealing a high degree of similarity ($r > 0.95$).

(**H**) Left: spike raster, and the mean FR over 10 trials, respectively, of an example optotagged FS-PV neuron responding with increased spiking to 40 Hz blue light (473 nm, 1 s, 3 ms light pulses, left). Right: spike raster, and the mean FR over 10 trials, respectively, of a concurrently recorded WS neuron showing decreased spiking in response to light-activation of FS-PV neurons.

(**I**) Top: mean spontaneous waveform of the three neuron types. Bottom: comparison of the mean FS-PV waveform (n = 13 FS-PV neurons) to the mean waveform of individual FS-PV (red), NS (blue), and WS (gray) neurons, respectively. One dot = one neuron; black horizontal line: mean.

(**J**) The mean relative LFP power (2-100 Hz) during baseline and in response to light stimulation at 10 (left), 20 (middle), or 40 Hz (right). All stimulation frequencies increased the LFP power around the stimulated frequency and its harmonics. For number of recordings, see (K).

(**K**) The relative LFP power ratio in response to 10, 20, and 40 Hz activation of mPFC FS-PV neurons for all individual LFP recordings (1 recording site/session).

Scale bars: 500 µm (B); mean ± SEM (C, D, H, J).

FIGURE S3

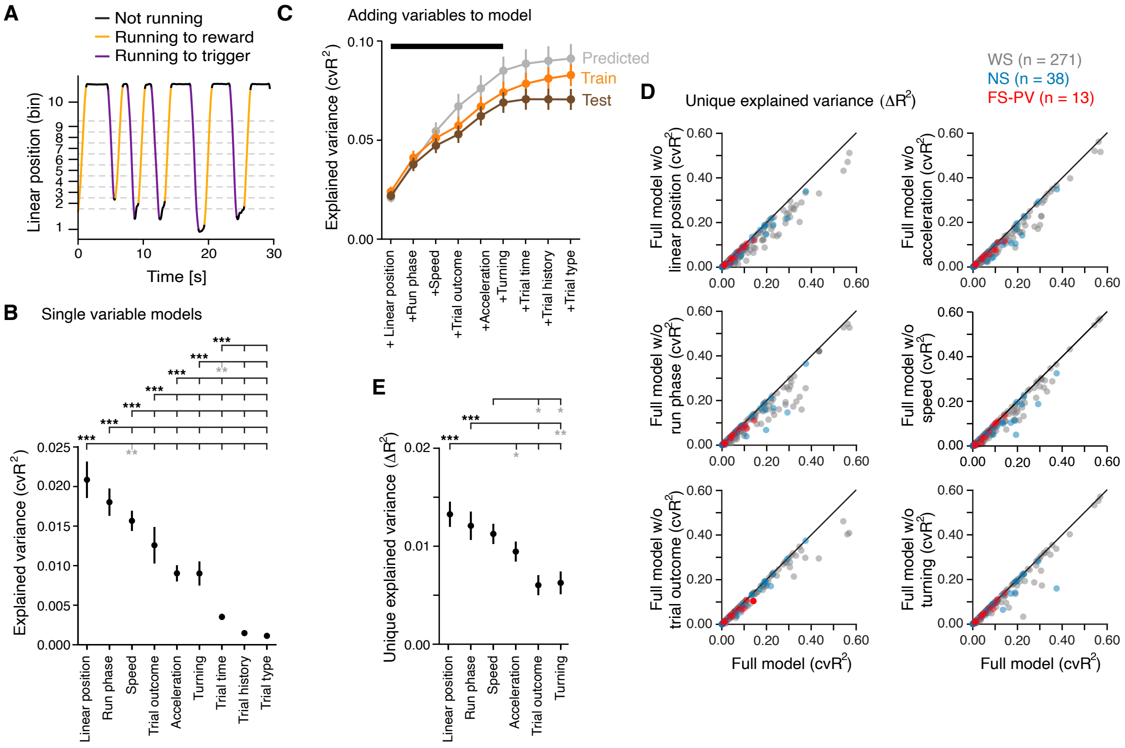

Figure S3. Variable selection for GLMs, related to Figure 2.

(**A**) Example scoring of the running behavior of one rat during 30s. During turning and reward consumption in the reward zone (part of bin 10) and during turning in the trigger zone (part of bin 1), the behavior is scored as ‘Not running’. The direction of the running (to reward vs to trigger) defines the variable run phase.

(**B**-**C**) Selection of behavioral and task variables for the GLMs.

(**B**) Explained variance (mean across neurons, n = 322), sorted in descending order, of linear models containing single behavioral or task variables.

(**C**) Explained variance (mean across neurons, n = 322) of linear models containing increasing numbers of variables. Brown: the explained variance of the test data, orange: the explained variance of the training data, gray: the cumulative sum of the explained variance of single variable models, representing the predicted explained variance if all variables are independent. The variables significantly increasing the explained variance of the test data (black horizontal bar) were included in the full model.

(**D**) Calculation of the unique explained variance (Δr^2) i.e., the difference between the explained variance of the full model (x-axis) and of the reduced models (y-axis). Scatter plots of the mean explained variance of the full models (x-axis) vs the respective reduced models (y-axis) for all individual neurons.

(**E**) The unique explained variance (mean across all neurons, n = 322) of the variables included in the full model.

Mean ± SEM (B, C, E); *$p < 0.05$, **$p < 0.01$, ***$p < 0.001$.

FIGURE S4

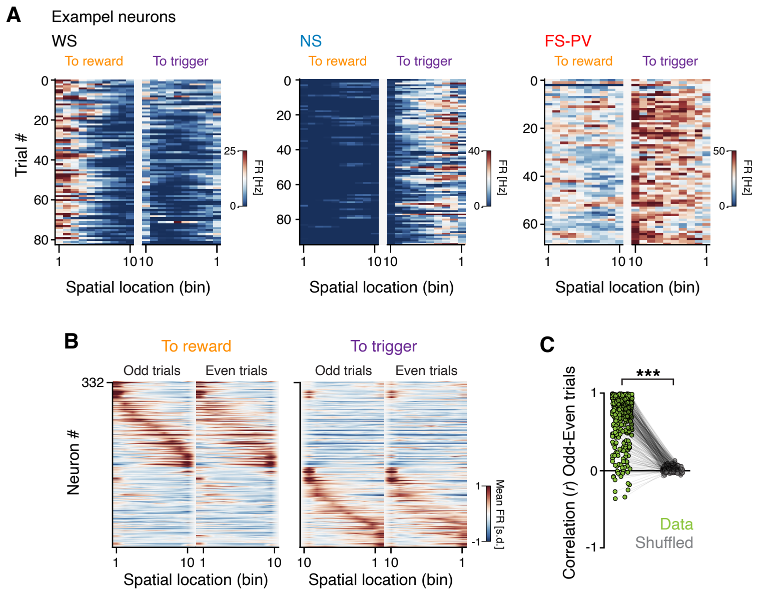

Figure S4. Consistent spatial representation across the PrL neurons, related to Figure 3.

(**A**) The FR in individual trials for one example neuron of each cell type.

(**B**) The trial-averaged FR (z-scored) of all individual neurons at the 10 track positions. The data was split into two groups based on the traversal direction (to reward vs to trigger), and each group was split into two based on odd vs even trial number. Neurons were sorted by the peak FR in odd trials and plotted in the same order for the even trials.

(**C**) The similarity of the FR between odd and even trials (Pearson's correlation (r)) was significantly higher for the real data compared to the shuffled data (circular shifted spike trains; $p = 5.1\times10^{-138}$, paired t-test).

***p < 0.001.

FIGURE S5

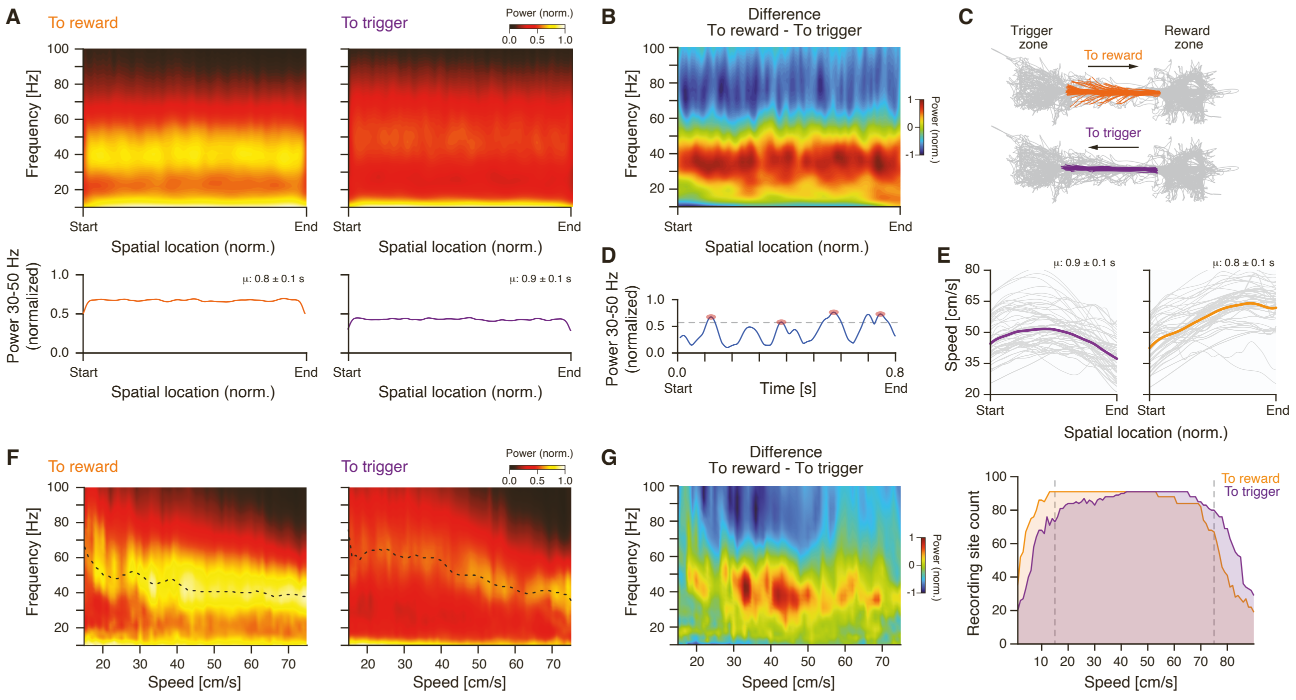

Figure S5. The modulation of PrL gamma oscillations during reward seeking is not related to speed processing, related to Figure 5.

(**A**-**G**) Analysis of the modulation of the LFP activity during the animals' running on the track, i.e., bins 2-9 or 9-2 (see Figure 2A). Only temporally homogeneous track traversals (0.5-1.5 s) are included in the analysis.

(**A**) Top: the mean power (normalized) in the LFP frequency spectrum (10-100 Hz) during traversals to reward (left) and to trigger (right), respectively. Bottom: extraction of the mean power in the 30-50 Hz gamma band. μ = mean traversal time.

(**B**) The difference in LFP power (normalized, 10-100 Hz) across the linear track between traversals to trigger and traversals to reward.

(**C**) Movement trace based on point at center of the head (gray) of one rat during a single session, tracked by DeepLabCut. Orange: detection of the rat's position on the linear track (bin 2-9; Figure 2A) during traversal to the reward platform; purple: detection of the rat's position on the linear track (bin 9-2; Figure 2A) during traversal to the trigger platform.

(**D**) Example detection of gamma bursts (red dots) in a single LFP trace (0.8 s) band-pass filtered on 30-50 Hz. Dashed line: the peak detection threshold (75th percentile power).

(**E**) Top: the running speed during track traversals to trigger (left) and to reward (right). Gray lines: the mean speed in individual sessions. Bottom: The distribution of speeds detected in the 91 recording sites (speed bin size = 1 cm/s). To adjust for the differential speed in the two track traversals, only speed bins within both traversals' speed distribution (15-75 cm/s, gray dashed vertical lines) were included in the LFP analysis (Figures 5G, S5F, G).

(**F**) The relationship between the mean LFP power (normalized, 10-100 Hz) and the running speed (15-75 cm/s) in traversals to reward (left) and to trigger (right). Dotted line: the frequency (30-80 Hz) with the maximum power in each speed bin.

(**G**) The difference in the relationship between the LFP power (normalized, 10-100 Hz) and the speed between traversals to trigger and traversals to reward.

47 sessions (A, B, E top); 91 LFP recording sites (A, B); LFP recording sites: To trigger n = 87 ± 4, To reward n = 88 ± 6 (mean ± s.d.; E bottom, F, G); Start/end = bin 2 or 9, depending on the traversal direction (see Figure 2A); mean ± SEM (A, bottom).
